# Effect of Acrylate Emulsion on the Mechanical and Microscopic Properties of Straw Fiber-Reinforced Cement-Magnesium Slag Stabilized Soil

**DOI:** 10.3390/polym16243462

**Published:** 2024-12-11

**Authors:** Chunqiu Xia, Xuanhao Cao, Jiuran Wen, Jun Li, Li Dai, Bowen Guan

**Affiliations:** 1 School of Materials Science and Engineering, Chang’an University, Xi’an 710061, China; 2022131015@chd.edu.cn (C.X.); caoxh@chd.edu.cn (X.C.); jun_li1992@163.com (J.L.); 2 Jiangxi Communications Investment Group Co., Ltd., Nanchang 330108, China; dlwhut2012@163.com

**Keywords:** soil stabilization, cement, magnesium slag, acrylate emulsion, microanalysis

## Abstract

In order to investigate the mechanism of mechanical performance enhancement and the curing mechanisms of acrylate emulsion (AE) in cement and magnesium slag (MS) composite-stabilized soil (AE-C-M), this study has conducted a comprehensive analysis of the compressive strength and microstructural characteristics of AE-C-M stabilized soil. The results show that the addition of AE significantly improves the compressive strength of the stabilized soil. When the AE content is 0.4%, the cement content is 3%, and the magnesium slag content is 3% (AE4-C3M3), the strength of the formula reaches 4.21 MPa, which meets the requirements of heavy traffic load conditions in the construction of high-speed or main road base layers. Some reactive groups on the polymer side chains (-COOH) engage in bridging with Ca^2+^ and RCOO^−^ to form a chemically bonded interpenetrating network structure, thereby enabling the acrylate emulsion to enhance the water damage resistance of the specimens. The notable improvement in strength is attributed to the film-forming and solidifying actions of AE, the binding and filling effects of C-S-H gel, and the reinforcing effect of straw fibers. FT-IR and TG-DSC analysis reveals the presence of polar electrostatic interactions between AE and the soil matrix. AE enhances the bonding among soil particles and facilitates the attachment of C-S-H gel onto the surfaces of the straw fibers, thereby increasing the strength and toughness of the material. The application of MS in conjunction with straw fibers within polymer-modified stabilized soil serves to promote the recycling of waste materials, thereby providing an environmentally friendly solution for the engineering application of solid waste.

## 1. Introduction

As a highly plastic clay that weathers into a reddish-brown or brownish-red hue under subtropical humid climatic conditions, red clay is characterized by its low natural density, high plasticity index, low compressibility, and considerable strength. Due to its favorable mechanical properties, it is often utilized in natural subgrades [1,2]. However, its susceptibility to poor water sensitivity poses significant challenges as it is greatly affected by water content; excessive moisture can lead to a mud-like consistency, while insufficient moisture predisposes it to cracking. Under high-temperature, dry climatic conditions, internal shrinkage resulting from water loss leads to the formation of cracks. During rainfall, water permeates through these cracks and is driven by capillary action into the clay matrix, thereby weakening the cementitious bonds [3,4]. To keep the soil stable, it is necessary not only to have a certain bearing capacity but also to have good resistance to erosion and frost heave and to have good adhesion and friction between soil particles, and this “expansion and contraction” behavior of clay poses a potential threat to the stability of the above engineering structures, resulting in severe incidents such as subgrade soil instability and sliding [5,6]. Such untreated plastic soils can pose a serious threat to the integrity of road subgrade engineering.

Cement-stabilized soil represents a highly effective soil improvement technique that is widely employed in the base and subbase layers of roads, benefiting from shorter construction periods [7,8]. However, cement-stabilized soil will cause the soil to become brittle to a certain extent, which may cause cracks in the process of heavy load, temperature change, and drying shrinkage, and may affect the structural performance. Researchers typically employ mineral admixtures to partially replace cement, thereby reducing material costs and carbon emissions. Xiaowen Xu et al. [9] incorporated phosphogypsum into the cement stabilized base of asphalt concrete pavement to explore the potential reuse possibility of phosphogypsum. The study showed that energy consumption and carbon emissions were reduced by 48.0% and 67.5% in the construction stage. Magnesium slag (MS), an industrial byproduct generated during the Pidgeon process for magnesium production, results in 5 to 10 tons of reducing slag for every ton of metallic magnesium produced [10,11]. Presently, the resource utilization rate of MS in China is approximately 30%. It is noteworthy that the mineral phases of MS are similar to those of cement, primarily consisting of β-C_2_S, SiO_2_, and CaO. Therefore, utilizing MS to partially replace cement in cement-stabilized soil emerges as a feasible and effective method for the treatment of solid waste [12,13,14]. Additionally, fibers are often incorporated into cement-stabilized soil to enhance soil reinforcement more effectively. Research has demonstrated that fiber-reinforced stabilized soil layers ensure isotropic strength stability and significantly minimize the likelihood of fault formation in the reinforcement direction [15,16]. Compared to unreinforced soil, fiber-reinforced soil exhibits improved ductility, with no reduction in post-peak strength. However, the commonly used synthetic fibers are associated with relatively high costs and limited applicability. The substitution of synthetic fibers with straw fibers in cement-stabilized soil not only contributes to sustainable development but also provides a solution for the reutilization of agricultural waste.

It is noteworthy that cement-stabilized soil treated with fiber reinforcement tends to exhibit lower curing strength and dispersive resistance in humid and rainy environments [17]. Studies have indicated that the incorporation of polymer emulsions can improve the capillary behavior of the soil, thereby reducing moisture migration and maintaining structural performance [18]. Furthermore, due to the sealing effect of polymer films that diminish harmful porosity, the inclusion of polymers can also enhance the durability of cement-stabilized soil, such as its impermeability [19]. It can improve soil strength through the formation of bonds between polar end groups of clay particles and polymers [20]. Carboxyl (protonated) or carboxylic acid (ionized) groups are attached to the surface of latex particles through copolymerization with methacrylic acid (MAA), enabling the complexation and adsorption of heavy metal ions within the soil matrix [21]. In the research conducted by Baghini, it was discovered that the utilization of carboxylated styrene–butadiene emulsion resulted in an 81.5% increase in the flexural modulus of the composite material [22]. Furthermore, Liu Jin et al. employed polyurethane organic polymers (PU) and polypropylene fibers (PF) to reinforce sandy soils, considerably increasing the tensile strength of the stabilized sand [23]. The soil treated with acrylic emulsion has better performance in water resistance and erosion resistance; can effectively resist the degradation caused by rain erosion and environmental humidity changes; has relatively strong durability; can effectively resist natural factors such as freeze–thaw cycles and temperature changes and reduce the risk of damage to the roadbed; and is more environmentally friendly in the construction process [24,25,26]. As anticipated, the mere presence of fibers is insufficient to adequately reinforce sandy materials. Consequently, the introduction of polymers facilitates the formation of a coherent matrix within the soil. The combination of physical and chemical reinforcement can generate a more pronounced synergistic effect. However, despite the progress achieved in the application of polymers for soil stabilization, the synergistic mechanisms between environmentally friendly and high-performance acrylic emulsions and fiber-reinforced stabilized soil remain unclear. The interaction mechanisms between fibers and the clay interfacial layer under the influence of polymers have been inadequately studied. This lack of research significantly limits the further application of acrylic emulsions in fiber-reinforced cement-stabilized soil.

In view of this, the present study investigates the impact of acrylate emulsion on the mechanical properties of straw fiber-reinforced cement–MS-stabilized soil (AE-C-M) through unconfined compressive strength testing. The characteristics of strength loss of the specimen were analyzed through the dry–wet cycles test to explore the quality of its water stability. Additionally, the interaction mechanisms and stabilization principles between acrylate molecular chains and fibers or clay particles are analyzed using XRD, FT-IR, TG-DSC, and SEM techniques. By investigating the synergistic mechanisms among C-S-H gel cementation hardening, the reinforcement treatment with straw fibers, and the polar interactions between AE curing agents and soil particles, the advancement of acrylate-incorporated straw fiber-reinforced cement–MS-stabilized soil in large-scale road engineering applications can be facilitated.

## 2. Materials and Methods

### 2.1. Materials

**Figure 1:**
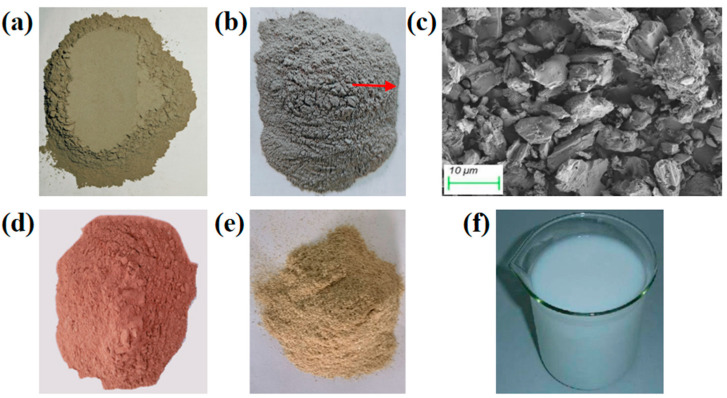
Appearance images of raw materials: (**a**) OPC; (**b**) MS; (**c**) SEM image of MS; (**d**) soil; (**e**) straw fiber; (**f**) acrylate emulsion.

**Figure 2:**
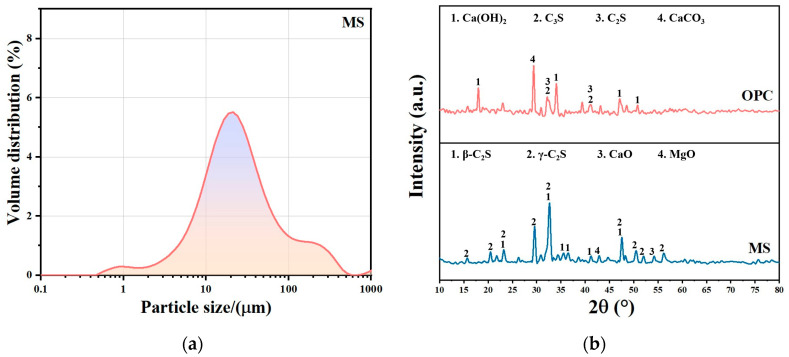
(**a**) Particle size distribution curve of MS. (**b**) The phase composition of OPC and MS.

The detailed chemical compositions and appearance characteristics of the raw materials used in this study are presented in Table 1 and Figure 1 (including SEM images of MS), respectively. In this research, red clay sourced from Jiangxi region of China was employed as the stabilizing substrate, and the test results of its technical indicators, conducted in accordance with “Testing Methods for Geotechnical Engineering in Highway Engineering” (JTG 3430-2020), are shown in Table 2. The cement utilized was P.O.42.5 ordinary Portland cement (OPC) purchased from Conch Cement (Xi’an, China), with its basic properties outlined in Table 3. Magnesium slag, which is a by-product originating from a specific smelting plant (Yulin, China), has been chosen as a partial replacement for ordinary Portland cement (OPC), and its characteristics, including particle size distribution and phase composition, are comprehensively illustrated in Figure 2. The straw fibers applied possess a length of 250 μm. The polymer emulsion used in this study was acrylic emulsion purchased from China Linxing Chemical (Xiangyang, China). Acrylate emulsion (AE) appears milky white, with a solid content of 47 ± 1 wt%, a pH range of 7–9, a viscosity of 1000 ± 50 mPa·s, and a glass transition temperature of 25 °C.

### 2.2. Specimen Preparation

The mixture proportions of the specimens are presented in Table 4. Compaction tests were conducted in accordance with the “Testing Methods for Geotechnical Engineering in Highway Engineering” (JTG 3430-2020) T0131-2019 to determine the optimum moisture content and maximum dry density for each group of cement-stabilized soil. In the compaction process, the mixture is divided into four equal volumes in the compaction cylinder according to the quarter method, and the compaction is 90 times each time, and finally the demolding, sampling, and drying are carried out. The MS was screened using a standard sieve with a mesh size of 0.075 mm to control its fineness. The water-soluble acrylate emulsion is adjusted to the optimal water content by mixing it in water at a rate of 60 r/min on a magnetic stirrer for 10 min, then it is mixed with loess for 8 h stew so that the acrylic emulsion and soil particles are fully infiltrated, and then the loess is mechanically mixed with cement/MS and straw fiber for 15 min. The specimen fabrication method was conducted in accordance with the “Test Procedures for Inorganic Binder Stabilized Materials in Highway Engineering” (JTGE51-2009). For the production of non-lateral compression specimens, the diameter × height = Φ50 × 50 mm test mold was selected according to the standard of fine grain soil. The loading rate of 1 mm/min was pressed on the TYE-2000B pressure test machine, and the pressure was maintained for 2 min. Then, the samples were demolded and put into the curing box.

### 2.3. Test Methods

**Figure 3:**
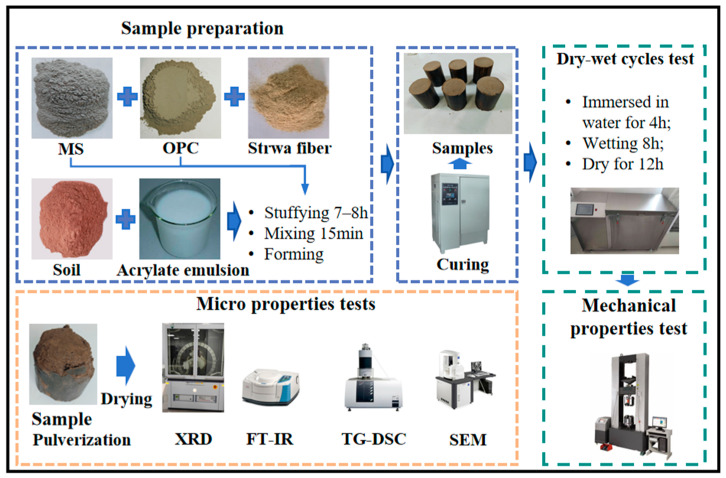
Sample preparation and performance testing process.

The entire experimental process of this study encompasses the preparation steps for AE-C-M composite-stabilized soil and the testing protocols. Unconfined compressive strength tests were conducted on the fabricated specimens. To investigate the strength development patterns and formation mechanisms of AE-C-M, microstructural performance assessments were performed on the samples. The comprehensive experimental procedure is illustrated in Figure 3.

#### 2.3.1. Mechanical Properties Test

The unconfined compressive strength tests of AE-C-M stabilized soil were conducted using a compressive and flexural testing machine, with the loading rate set at 1 mm/min. The specimens utilized were cylindrical, with a diameter of 50 mm and a height of 50 mm. Each group of specimens consisted of six samples, and the arithmetic mean of these samples was adopted as the final experimental strength.

#### 2.3.2. Dry–Wet Cycles Test

According to highway geotechnical test standard JTG 3430-2020, dry and wet cycle tests were carried out on the samples. The dry–wet cycling experiment encompasses both the immersion phase and the drying phase. For the AE-C-M specimens, a soaking method is employed for moisture enhancement treatment. The complete dry–wet cycle consists of the sequence of moisture absorption followed by drying. The specimens are wrapped with absorbent paper, and water is gradually added until they are fully submerged. Considering the weak water stability characteristics of the stabilized soil, the immersion duration is controlled to 4 h, followed by a continuous wetting process using absorbent paper for an additional 8 h. The drying process involves the placement of the air-dried specimens into an oven for further drying treatment. The drying temperature is maintained at 20 °C, and the drying duration is regulated to 12 h. Based on the fundamental physical properties of the soil, the number of dry–wet cycles is set to 1, 2, 4, 6 cycles. Upon the conclusion of the wet–dry cycles, mechanical performance tests are conducted once more, employing the strength loss rate to analyze the water stability performance of polymer-modified stabilized soil.

#### 2.3.3. Micro Properties Tests

To investigate the mechanism of structural strength formation and the microstructural characteristics of AE-C-M, powder samples were subjected to X-ray diffraction (XRD) analysis using a Smartlab SE, which facilitated the examination of the phase composition of the AE-C-M stabilized soil. The scan rate was established at 10°/min, and the scanning angle range was set from 10° to 80°. Fourier-transform infrared spectroscopy (FT-IR) was conducted using a Thermo Nicolet iS5 (Waltham, MA, USA) in attenuated total reflectance (ATR) mode with 32 scans performed on the samples. The spectral range was recorded from 400 cm^−1^ to 4000 cm^−1^, with a resolution of 4.00 cm^−1^. A TA-SDT650 synchronous thermal analyzer was employed for thermogravimetric–differential thermal analysis (TG-DSC) of the samples, wherein the ground and dried powdered samples were placed into the sample pan. The temperature range for the experiment was set from 25 °C to 800 °C, with a heating rate of 10 °C/min, using nitrogen as a protective gas to prevent sample carbonization. Additionally, scanning electron microscopy (ZEISS Sigma 300, Oberkochen, Germany) was utilized to analyze the microstructural morphological characteristics of the samples.

## 3. Results and Discussion

### 3.1. Mechanical Properties

**Figure 4:**
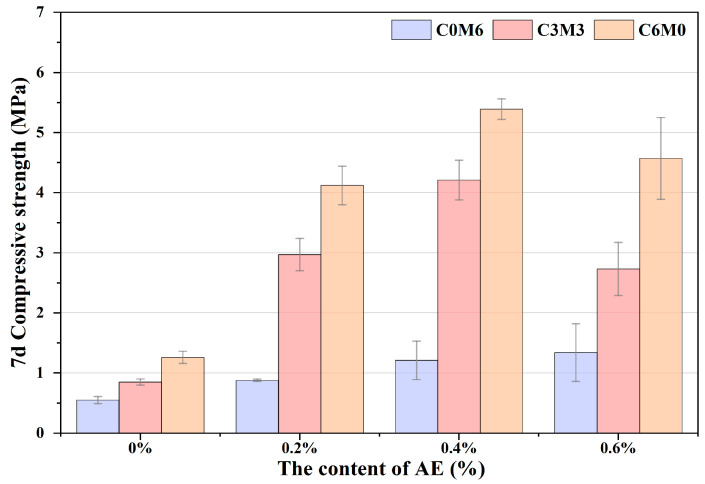
7d Compressive strength of AE-C-M stabilized soil.

The results of the unconfined compressive strength tests for the AE-C-M stabilized soil after 7 days are illustrated in Figure 4. A notable trend is observed wherein the compressive strength of the specimens initially increases significantly with the rising dosage of AE, followed by a slight decrease. This significant increase in strength can be attributed to the binding and curing effect of calcium silicate hydrate (C-S-H) gel, the strengthening effect of straw fiber, in addition to the film forming effect of acrylate emulsion, which generates a continuous and uniform film on the surface of soil particles. These polymer films have good bonding properties and form strong chemical and physical bonds between soil particles, enhancing the overall strength and stability of the soil. The impact of AE on the strength enhancement of the soil matrix is particularly striking, with the maximum compressive strength of the AE4-C6M0 specimen reaching 5.39 MPa, which represents an increase of over three times compared to the AE0-C6M0 group. This level of strength is wholly sufficient to meet the requirements for heavy traffic loads in high-speed or first-class road base applications. It is noteworthy that when the dosage of AE reaches 0.6‰, the 7-day compressive strength of the specimens decreases to 4.57 MPa. This phenomenon may be associated with the ionization of carboxyl groups within the AE, resulting in electrostatic repulsion of OH^−^ ions around the soil particles, complicating the formation and expansion of the gel membrane necessary for strength development [27,28]. This also indicates that the hydration reaction in soil matrix leads to the high content of OH^−^, which is unfavorable to the dispersion of AE components and thus inhibits the film forming effect [29]. Importantly, the substitution of 50% of the cement with MS did not lead to a significant reduction in strength. In fact, the strength of the AE4-C3M3 composition even exceeded that of the AE2-C6M6 composition, reaching 4.21 MPa, thus satisfying the demands for heavy traffic loads in the construction of highways and arterial roads. The selection of the C3M3 group under the condition of 0.4‰ AE not only meets the strength requirements but also promotes the resource reutilization of metallurgical waste, providing a feasible implementation strategy for future solid waste road engineering applications.

### 3.2. Strength Characteristics Under Dry–Wet Cycles

**Figure 5:**
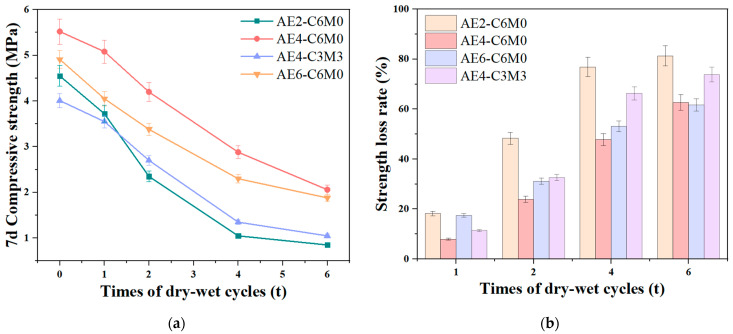
(**a**) Effect of dry–wet cycles on the 7d compressive strength of AE-C-M. (**b**) Strength loss rate of AE-C-M under dry–wet cycles.

Four experimental groups were selected to conduct wet–dry cycle tests in order to further investigate the impact of acrylate emulsion on the strength properties of straw fiber-reinforced cement-stabilized soil under wet–dry cycling conditions, as illustrated in Figure 5. In Figure 5a, it is distinctly observed that with the increasing number of wet–dry cycles, the compressive strength of the specimens exhibited a significant reduction. It is noteworthy that the strength loss under wet–dry cycling conditions showed considerable variability among the different experimental groups. As shown in Figure 5b, after six wet–dry cycles, the total strength loss of AE2-C6M0 reached 81.32%, while that of AE6-C6M0 was only 61.71%. This indicates that the increased dosage of the acrylate emulsion led to a reduction in the strength loss of the stabilized soil specimens. This phenomenon may be attributed to the polymer films formed by the acrylate emulsion, which effectively hinder moisture from eroding the internal structure of the soil [30]. Additionally, some reactive groups on the polymer side chains, such as -COOH, may react with hydration products of cement, including Ca^2+^ and Al^3+^, forming a chemically bonded interpenetrating network structure through the bridging of large molecules by Ca^2+^ and RCOO^−^ [31]. This allows for the absorption of some internal stresses generated by drying shrinkage, thereby reducing the formation of weakened interfacial gaps and alleviating the instantaneous strength loss induced by wet–dry cycling [32,33,34]. It is also important to note that AE4-C6M0 exhibited relatively low strength loss rates under conditions not exceeding six wet–dry cycles, with an initial strength loss of only 7.97%. This may be because excessive AE content may lead to uneven distribution of the polymer in the soil, thus affecting the overall structural stability of the soil, adversely affecting its strength and durability. Additionally, AE4-C3M3 demonstrated robust water damage resistance during the first two wet–dry cycles, with a strength loss of only 32.67% after the second cycle, second only to AE4-C6M0. Although the replacement of 50% of cement with MS resulted in a decreased cementation effect among the soil structure, the excellent dispersion of 0.4‰ acrylate emulsion within the clay matrix imparted a certain degree of water damage resistance to the specimens.

### 3.3. XRD Analysis

**Figure 6:**
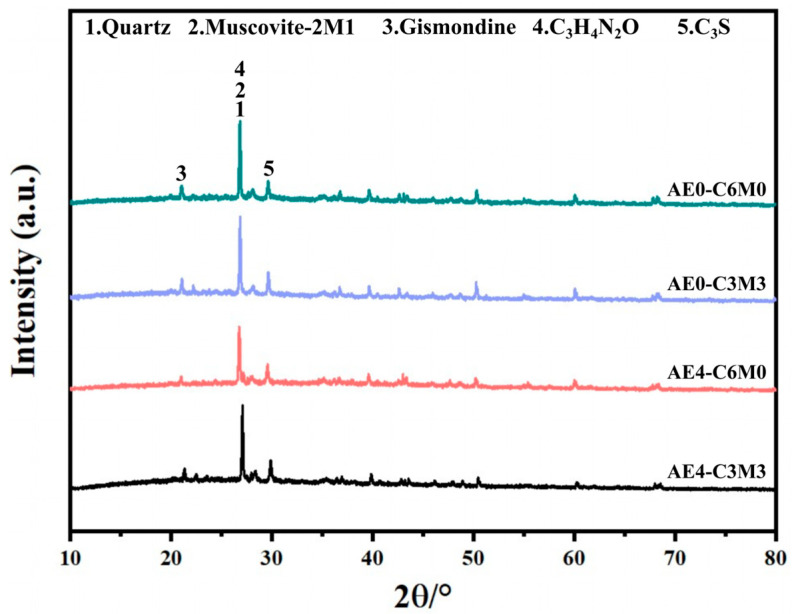
XRD spectrum of AE-C-M stabilized soil.

The XRD test results for the AE-C-M stabilized soil are presented in Figure 6. Several significant phase compositions are discerned within the XRD spectrum, including quartz, muscovite-2M1, gismondine, and the C_3_H_4_N_2_O component. Notably, the crystallinity of the C-(A)-S-H gel phase is relatively low, which precludes the manifestation of diffraction peaks within the spectrum. Among the observed diffraction peaks, the one at 2θ = 21.03° corresponds to gismondine, an aluminosilicate mineral that provides the necessary aluminum groups for the transformation of C-S-H gel phase into a high-strength C-(A)-S-H gel, representing a vital constituent of clay minerals [35]. The diffraction peak located at 2θ = 26.83° corresponds to characteristic peaks of quartz and muscovite-2M1. Silicon exists in the stabilized soil in the form of quartz, while muscovite-2M1 demonstrates considerable diffraction intensity as a component within the soil matrix [36]. The diffraction peak at 2θ = 29.65° corresponds to C_3_S, which is a principal mineral component of cement and undergoes hydration reactions to generate C-S-H gel with binding and hardening capabilities [37]. Furthermore, it can be observed that the incorporation of AE has a minimal impact on the diffraction peak intensity of the products within the system, likely attributable to the low purity and content of the products. Importantly, the diffraction peak for C_3_H_4_N_2_O appears exclusively in the samples containing AE [38]. This phenomenon may be attributed to the reaction between the carboxyl groups in the acrylic molecular chains and the amino groups present in the soil, which relates to the film-forming and curing actions of acrylic acid within the soil matrix.

### 3.4. FT-IR Analysis

**Figure 7:**
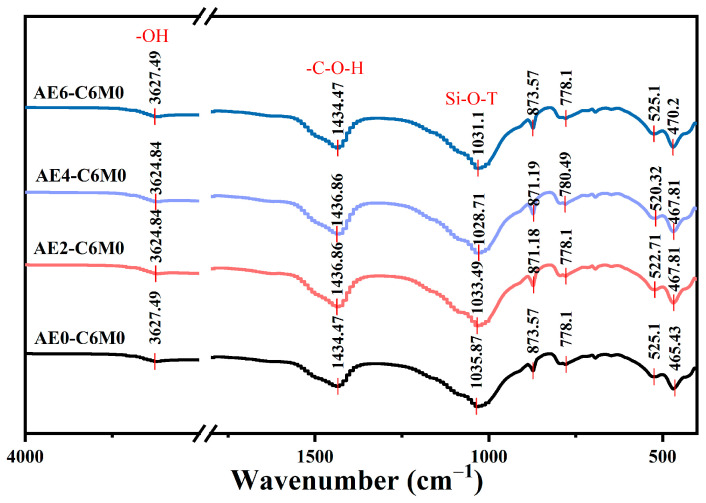
FTIR spectra of AE-C-M stabilized soil.

The FTIR spectra of the AE-C-M stabilized soil are illustrated in Figure 7, from which three prominent vibrational absorption bands can be distinctly observed at 1434 cm^−1^, 1035 cm^−1^, and 467 cm^−1^. Additionally, the characteristic peak at 3627 cm^−1^ results from the stretching and bending vibrations of -OH bonds, associated with the typical stretching vibrations of hydroxyl groups in water molecules present within the soil matrix. The shift of -OH stretching vibrations towards lower wavenumbers indicates the occurrence of electrostatic interactions between the protonated functional groups (-OH) and cations existing in the soil matrix [39]. When an excessively high dosage of AE is applied, a tendency towards the restoration of lower intensity stretching vibrations is noted, attributed to the ionization of carboxyl groups in the surplus AE molecules which then generated anionic species; this phenomenon subsequently led to a degree of electrostatic repulsion with the OH^−^ groups present in the soil [40]. The peak observed at 1434 cm^−1^ is due to the stretching vibrations of the -C-O-H groups, with the fluctuations correlated to the increasing dosage of AE. The hydration reactions of cement/MS and other cementing materials produce a substantial amount of OH^−^, which can induce an alkaline environment within the soil, thus facilitating the hydrolytic condensation of AE in the alkaline soil matrix. This effect may also be related to the film-forming process of the acrylic molecules within the soil structure [41,42]. Furthermore, during the developmental phase, unique new vibrational peaks emerge at 873 cm^−1^ and 778 cm^−1^, and the stretching vibrations of these new peaks are potentially associated with the adsorption of carboxyl groups onto soil particles. Lastly, the stretching vibrations of the Si-O-T (T = Si/Al) groups are observed at 1035 cm^−1^, which are notably related to the changes in chain length of silicate tetrahedra caused by the presence of C-S-H gel within the soil. The shift of this asymmetric stretching vibration peak towards lower wavenumbers may suggest the presence of external adsorption forces or the formation of bonds that enhance the stability of Si-O-T groups [43]. It can be inferred that the structure of AE-C-M comprises an organic phase (i.e., AE) and an inorganic phase (i.e., loess), resembling the complexation behavior of AE-clay within soil particles. 

### 3.5. TG-DSC Analysis

**Figure 8:**
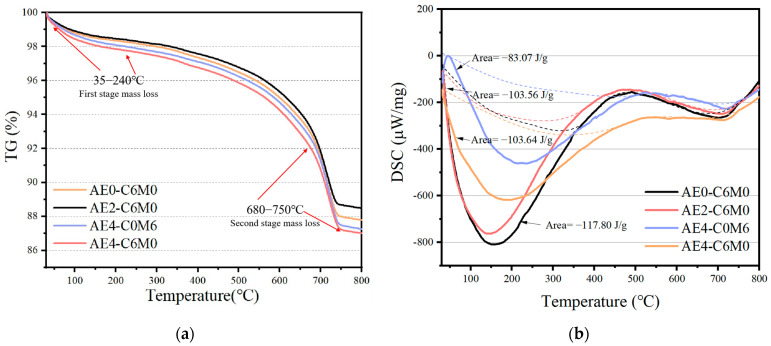
TG and DSC curves of AE-C-M stabilized soil: (**a**) TG curve; (**b**) DSC curve.

The hydration process of the internal structure of AE-C-M was further analyzed through TG-DSC analysis, as illustrated in Figure 8. It is evident that the thermal decomposition curves of the four sets of AE-C-M samples indicate that the incorporation of AE did not significantly alter the hydration progress of AE-C-M. Within the TG curve, two distinct temperature ranges for thermal decomposition can be observed, corresponding to the two endothermic peaks in the DSC curve.

(I) In the temperature range of 35–240 °C, the weight loss is primarily attributed to the evaporation of products such as ettringite (AFt) and C-S-H gel formed during the hydration reactions of cement. Additionally, the dehydration of AFm crystals occurs at approximately 100 °C, with further dehydration observed around 120 °C. Notably, most of the water evaporating during this stage exists predominantly in a free state [44].
(II) In the temperature range of 680–750 °C, the endothermic peak in this stage is generated by the thermal decomposition of CaCO_3_, mainly as a result of the carbonation of the samples during the curing process, with a significant increase in weight loss observed as the curing time is extended [45].

It is also noteworthy that the substantial weight loss observed between 300 °C and 700 °C may be attributed to the thermal decomposition of silicate and aluminate phases within the soil particles of AE-C-M. The integration results of the endothermic peak areas from the DSC curves indicate that AE0-C6M0 exhibits the highest endothermic peak intensity (Area = −117.80 J/g), while AE4-C0M6 demonstrates a substantially lower trend (Area = −83.07 J/g). This phenomenon can be attributed to the lower reactivity of the MS in the system compared to that of cement, resulting in a reduced quantity of C-S-H gel generated during the hydration process, which consequently leads to a lower upper limit of endothermic heat absorption during thermal decomposition [46]. Importantly, the temperature of the endothermic peaks for the experimental group incorporating 0.4‰ AE is observed to be higher than that of the group without AE incorporation. This indicates that the inclusion of AE enhances the thermal stability of the AE-C-M system. Such a phenomenon is likely related to the film-forming and hardening actions of AE within the system. Consequently, significant reductions in the thermal decomposition rates of AE4-C6M0 and AE4-C0M6 are observed between the temperatures of 30 °C and 221.44 °C. Furthermore, the alkalinity generated within the system during the hydration process of the cementing materials induces changes in both the chemical composition and crystalline structure of the polymers within the soil particles [47]. The observed decrease in thermal decomposition rates of the experimental groups with increasing AE dosage further suggests the occurrence of interactions and film-forming effects between the acrylic acid and aluminosilicate phases within the soil matrix during the thermal decomposition of acrylic acid, thereby inhibiting the weight loss processes associated with the crystalline water and decomposition of acrylic acid within the structure [48].

### 3.6. SEM Analysis

**Figure 9:**
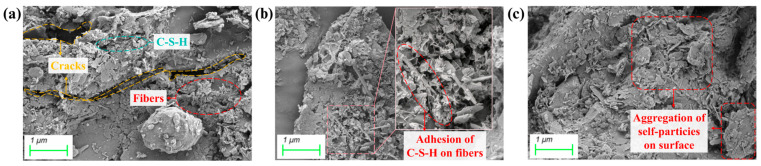
SEM images of AE-C-M stabilized soil: (**a**) AE0-C6M0; (**b**) AE4-C6M0; (**c**) AE4-C0M6.

The results of the SEM tests for the AE-C-M stabilized soil are presented in Figure 9. The mechanism underlying the strength enhancement of the AE-C-M stabilized soil is based on the film-forming and solidifying actions of AE on soil particles, as well as the effective adhesion of C-S-H gel to the surface layers of the straw fibers. In Figure 9a, a significant presence of interface cracks and voids can be clearly observed in the structural region of AE0-C6M0, where a considerable number of straw fibers fails to exhibit effective reinforcement. Notably, in the structural region of AE4-C6M0, a marked roughening of certain fiber surface layers is identified, which facilitates the efficient adhesion and bonding of C-S-H gel generated from the hydration of cement and MS to the soil particles [49]. The roughening of the straw fiber surface layer is likely attributed to the interactions between the carboxyl groups present in the acrylic molecules and the OH^−^ ions produced during the hydration of cement, which may alter the surface characteristics of the fibers, rendering them rougher and irregular [50]. Such a rough surface can provide a greater number of mechanical interlocking points, thereby allowing for enhanced adhesion of C-S-H gel to the fiber surfaces during the curing process.

Additionally, in the AE4-C0M6 composition, a phenomenon of self-aggregation among soil surface has been observed, which can be attributed to the film-forming and solidifying effects of the acrylic molecules on the surface. Furthermore, the incorporation of straw fibers effectively suppresses the initiation of microcracks through reinforcing action, thereby improving the ductility and crack resistance of the soil matrix [51]. This reinforcement mechanism allows the material to better distribute stress under load, reducing the incidence of localized stress concentration. Moreover, the film-forming action of acrylic further elevates the crack resistance of the material [52]. The membrane formed by the acrylic within the soil acts to effectively impede rapid moisture evaporation, thereby mitigating the formation of cracks induced by drying. The presence of this membrane not only enhances the durability of the material but also improves its stability under adverse environmental conditions.

## 4. Conclusions

In response to the application environment of subgrade soil, this study puts forward stricter requirements on the resistance to dry–wet cycle and high bearing capacity of subgrade soil. After the stabilization of red soil by acrylic emulsion and fiber cement, its resistance to dry–wet cycle and bearing capacity fully adapt to the application range of subgrade soil in red soil areas. In this paper, the influence of acrylate emulsion on straw fiber cement/magnesium slag-stabilized soil was evaluated, and its microscopic mechanism was further analyzed. The conclusions are as follows:

(1) The incorporation of acrylic ester emulsion (AE) significantly enhances the compressive strength of straw fiber-reinforced cement–MS stabilized soil. An optimal amount of AE, such as 0.4‰, maximally increases soil strength, while excessive AE, such as 0.6‰, may reduce strength due to electrostatic repulsion. Notably, when MS replaces 50% of the cement, soil strength is preserved, with the AE4-C3M3 formulation meeting strength requirements for high-load conditions on expressways and primary roads.
(2) Under wet–dry cycling conditions, the acrylate emulsion is capable of enhancing the water damage resistance of straw fiber-reinforced cement-stabilized soil. The total strength loss of AE2-C6M0 reached 81.32%, while the total strength loss of AE6-C6M0 is limited to only 61.71%.
(3) The film-forming action of the acrylate emulsion enables the absorption of a portion of the drying shrinkage stress, subsequently reducing the instantaneous strength loss induced by wet–dry cycling. The variability in strength loss rates observed under fewer than six wet–dry cycles indicate the existence of an optimal dosage of acrylate emulsion (0.4‰).
(4) The interaction mechanism between the groups in AE-C-M has been elucidated through FTIR analysis. The addition of AE shifts the -OH stretching vibration to lower wavenumbers, indicating electrostatic interactions with cations in the soil matrix. Variations in the stretching vibrations of -C-O-H groups have also been observed, likely related to the hydrolysis and condensation processes of AE in the alkaline soil matrix. The emergence of new absorption peaks at 873 cm^−1^ and 778 cm^−1^ indicates the adsorption of AE’s carboxyl groups onto soil particles, enhancing soil stability.
(5) The increase in strength results from the film-forming and solidifying actions of AE, the bonding of C-S-H gel with soil particles, and the reinforcing effects of straw fibers. AE promotes self-aggregation of soil particles, while the rough surfaces of straw fibers facilitate C-S-H gel adhesion, improving the toughness and crack resistance of the soil matrix.

## Figures and Tables

**Table 1:** Chemical composition of the soil and MS (wt.%).

Material	CaO	SiO_2_	Al_2_O_3_	Fe_2_O_3_	SO_3_	MgO	K_2_O	Na_2_O	Other
Soil	58.87	18.49	5.33	5.08	2.68	2.08	0.74	0.19	6.54
MS	60	27	1.2	7.4	0.1	3.3	0.1	0.09	0.81

**Table 2:** Basic physical properties of soil.

Material	Natural Moisture Content/%	Plastic Limit/%	Liquid Limit/%	Plasticity Index/I_p_	Maximum Dry Density/(g/cm^3^)	Optimum Water Content/w_op_
Soil	10.21	7.87	26	18.13	1.838	13.72%

**Table 3:** Basic physical properties of OPC.

Material	SiO_2_	Al_2_O_3_	CaO	Fe_2_O_3_	MgO	K_2_O	TiO	Na_2_O	Other
P.O.42.5	18.49	5.33	58.87	5.08	2.08	0.74	0.25	0.19	4.77

**Table 4:** Mixture ratio of AE-C-M composite material.

Sample	OPC (%)	MS (%)	Acrylate Emulsion (‰)	Straw Fiber (%)
AE0-C6M0	6	0	0	0.4% (all samples)
AE0-C3M3	3	3		
AE0-C0M6	0	6		
AE2-C6M0	6	0	0.2	
AE2-C3M3	3	3		
AE2-C0M6	0	6		
AE4-C6M0	6	0	0.4	
AE4-C3M3	3	3		
AE4-C0M6	0	6		
AE6-C6M0	6	0	0.6	
AE6-C3M3	3	3		
AE6-C0M6	0	6		

## Data Availability

Data are contained within the article.
